# Microwave-Assisted Synthesis of Carbon-Based (N, Fe)-Codoped TiO_2_ for the Photocatalytic Degradation of Formaldehyde

**DOI:** 10.1186/s11671-015-1061-6

**Published:** 2015-09-16

**Authors:** Fei Tian, Zhansheng Wu, Yanbin Tong, Zhilin Wu, Giancarlo Cravotto

**Affiliations:** School of Chemistry and Chemical Engineering, Shihezi University, Shihezi, 832003 People’s Republic of China; Dipartimento di Scienza e Tecnologia del Farmaco, University of Turin, Turin, 10125 Italy

**Keywords:** Photocatalytic degradation, Microwave irradiation, Active carbon, Codoped AC/TiO_2_, Formaldehyde

## Abstract

A microwave-assisted sol–gel method was used to synthesize (N, Fe)-codoped activated carbon (AC)/TiO_2_ photocatalyst for enhanced optical absorption in the visible light region. The prepared samples were characterized via X-ray diffraction, scanning electron microscopy, transmission electron microscopy, Fourier transform infrared spectroscopy, Brunauer–Emmett–Teller analysis, ultraviolet–visible light spectroscopy, X-ray photoelectron spectroscopy, and photoluminescence spectroscopy. The results showed no significant difference in the surface area of AC/TiO_2_ (approximately 500 m^2^/g) after doping. TiO_2_ was uniformly distributed on the surface of AC, which exhibited coexisting anatase and rutile structures with a mean crystallite diameter of approximately 20 nm. N and Fe monodoping on AC/TiO_2_ reduced the energy band gap of TiO_2_ to 2.81 and 2.79 eV, respectively, which mainly attributed to the impurity energy formed in the energy gap of TiO_2_. In (N, Fe)-codoped AC/TiO_2_, N and Fe are incorporated into the TiO_2_ framework and narrow the band gap of TiO_2_ to 2.58 eV, thereby causing a large redshift. Codoping of N and Fe enhanced the production of hydroxyl radicals (⋅OH) and improved the photocatalytic activity of the resultant AC/TiO_2_ compared with those of undoped and N- or Fe-monodoped AC/TiO_2_. N-Fe-AC/TiO_2_ degraded 93 % of the formaldehyde under Xe-lamp irradiation. Moreover, the photocatalyst was easily recyclable. In summary, a novel and efficient method to mineralize low concentrations of HCHO in wastewater was discovered.

## Background

Formaldehyde (HCHO) is a volatile organic compound that irritates the respiratory, cardiovascular, and nerve tissues of humans [1]. Thus, HCHO removal is essential for improving environmental quality. HCHO photodegradation in the presence of a titanium dioxide (TiO_2_) photocatalyst completely degrades HCHO into CO_2_ and H_2_O [2]. Unfortunately, the photocatalytic activity of TiO_2_ is limited by its low adsorption property and large band gap (3.2 eV) [3, 4].

Significant efforts have been made to overcome these two drawbacks. Preparation of TiO_2_ photocatalysts loaded with porous materials and characterization of their photocatalytic performance have drawn great research attention [5]. Combination of the distinctive properties of mesoporous carbon materials and TiO_2_ evidently improves optical absorption and has been adopted in the removal of organic contaminants [6]. Pastravanu et al. [7] reported that 92 % conversion of methyl orange is achieved after 170 min of ultraviolet (UV) irradiation with AC/TiO_2_ composite, whereas only 42 % conversion is achieved with pure TiO_2_. However, in our previous studies [8], the degradation efficiency of AC/TiO_2_ (1 g) was only 36 % under UV irradiation for 420 min at a low concentration of HCHO (30 mg/L). Therefore, it is important to improve the photocatalytic performance of TiO_2_ by improving its internal structure. Metal or nonmetal doping of TiO_2_ could extend its optical absorption range into the visible light region and modify the generation rate of the electron–hole pairs [9–12]. TiO_2_ doped with transition metals has recently been prepared, and studies on these materials have shown that the energy band gap of TiO_2_ decreases with decreasing recombination rate of photogenerated electron–hole pairs [13–15]. Iron is considered one of the most appropriate transition metals for TiO_2_ doping because the atomic radius of Fe^3+^ is close to that of Ti^4+^, thus, the titanium positions in the TiO_2_ lattice can be easily replaced by iron cations [16–18], remarkably improving photocatalytic efficiency. The results of Safari et al. [19] showed significant improvements in the photodegradation of Reactive Orange 16 by Fe-doped TiO_2_ (nearly 93 %) compared with that by pure TiO_2_ (approximately 71 %) under UV irradiation. Doping TiO_2_ with nonmetallic anions, such as N, S, and C, has been proposed as a promising method for extending photoresponses from the UV to the visible light regions [20, 21]. Among them, N-doped TiO_2_ has been demonstrated to be the most effective in narrowing the band gap and increasing photocatalytic activity in the visible light region [22]. Several papers have reported the effects of Fe and N modification of TiO_2_ in enhancing photocatalytic activity [23, 24]. The photocatalytic activities of these powders are approximately two to four times higher than that of pure anatase TiO_2_ under visible light irradiation. In addition, using microwave irradiation to synthesize TiO_2_ nanoparticles is a recent innovation [25]. Compared with conventional methods, microwave irradiation presents several advantages in terms of cleanliness, short reaction times, and energy economy [26]. Because very few reports on the microwave synthesis of (N, Fe)-codoped TiO_2_ photocatalysts coated on AC (N-Fe-AC/TiO_2_) are available, the objective of the present work is to develop a rapid method to prepare N-Fe-AC/TiO_2_ and investigate its photocatalytic effect on HCHO under visible light irradiation.

The present work focuses on the synthesis of N-Fe-AC/TiO_2_ prepared using microwave irradiation and its structural characterization. The characteristics of the photocatalysts have been analyzed by X-ray diffraction (XRD), scanning electron microscopy (SEM), transmission electron microscope (TEM), Fourier transform infrared (FTIR), Brunauer–Emmett–Teller analysis (BET), ultraviolet and visible spectroscopy (UV–vis), X-ray photoelectron spectroscopy (XPS), and photoluminescence (PL). Studies on the use of catalysts for photodegradation of HCHO in aqueous solution under visible light irradiation are in progress.

### Experimental Methods

#### Catalyst Preparation

AC was prepared based on the precious study [27]. A mixture of solid KOH (10 g) and dried coal at a ratio of 1:1 was placed in a quartzose tube in a microwave reactor and activated under vacuum atmosphere at 693 W for 10 min. The obtained AC samples were pretreated by adding into HNO_3_ solution with 24 h. The mixture was filtered using distilled water until they became neutral. The pretreated AC was then dried and stored until use.

The TiO_2_ gel/sol was obtained by conventional sol–gel method. All reagents were of analytical grade and used without further purification. In typical synthesis process, 30 mL of tetrabutyl orthotitanate (TBOT) was dissolved in anhydrous alcohol (EtOH) in proportion of 1:1 (volume ratio). This solution was thoroughly stirred for 40 min and named solution A. Solution B was prepared by mixing 14 mL of glacial acetic acid and 7 mL of distilled water in 35 mL of absolute alcohol. Solution B was added to solution A dropwise and continuously stirred for 1 h. Then, it was obtained pale yellow clear TiO_2_ sol. Pretreated AC (10 g) was added into TiO_2_ sol (100 g). The mixture was placed in an oven at 100 °C for 24 h. After solidification, AC/TiO_2_ was prepared under microwave irradiation at 700 W for 15 min. To prepare Fe-doped AC/TiO_2_, Fe(NO)_3_⋅9H_2_O was mixed with solution B, while for N-doped AC/TiO_2_, urea was dissolved in solution B. The dosage of Fe iron was 0.008, 0.01, and 0.012 g, and the resulted samples were noted as 0.008 Fe-AC/TiO_2_, 0.01 Fe-AC/TiO_2_, and 0.012 Fe-AC/TiO_2_, respectively. The dosage of N was 0.2, 0.4, and 0.6 g, and the resulted samples were noted as 0.2 N-AC/TiO_2_, 0.4 N-AC/TiO_2_, and 0.6 N-AC/TiO_2_, respectively. Optimum concentrations of N and Fe were obtained by maximizing the photocatalytic activity for the monodoped (Fe or N) AC/TiO_2_. These optimized concentrations were used for synthesizing (N, Fe)-codoped AC/TiO_2_.

### Catalyst Characterization

The crystal structures of the prepared samples were measured through XRD on a Rigaku D/Max-2500/PC powder diffractometer. Each sample powder was scanned using Cu-*Kα* radiation with an operating voltage of 40 kV and an operating current of 200 mA. The surface micromorphologies of photocatalysts were characterized through SEM (S4800, Hitachi LTD) at an accelerating voltage of 15 kV. TEM was performed on a Tecnai G2 F20 microscope at 100 kV. FTIR spectra were recorded with a Bruker Vertex FTIR spectrometer, resolution of 2 cm^−1^, in the range of 4000–400 cm^−1^ by KBr pellet technique. The UV–vis DRS were obtained with a powder UV–vis spectrophotometer (U-4100, Hitachi LTD). Specific surface area (SBET, m^2^ · g^−1^) was calculated using the BET equation, and total pore volume (*V*_*t*_, m^3^ · g^−1^) was evaluated by converting the adsorption amount at *P*/*P*_0_ = 0.95 to the volume of liquid adsorbate. XPS analysis of samples was conducted using a PHI5700 ESCA system equipped with a Mg *Kα* X-ray source (1253.6 eV) under a vacuum pressure <10^−6^ Pa. The formation rate of ⋅OH at photo-illuminated sample/water interface was detected by the PL technique using terephthalic acid (TA) as a probe molecule. PL spectroscopy of synthesized products was taken at room temperature on a Hitachi F2500 spectrofluorometer using a Xe lamp with an excitation wavelength of 325 nm.

### Photocatalytic Activity

The photocatalytic activity of prepared photocatalyst was measured by degrading of the HCHO solution. In a typical test, 0.05 g of catalyst was added to 50 mL of HCHO solution (30 mg/L, pH = 6.8). The mixture was then irradiated under Xe lamp to degrade HCHO. The distance between the reactor and lamp housing is 8.5 cm. The removal efficiency of the photocatalyst can be calculated as follows:

$$ \eta =\frac{C_o-{C}_t}{C_o}\times 100\ \% $$

where *C*_o_ and *C*_*t*_ are the concentrations of HCHO at initial and different irradiation times, respectively.

## Discussion

**Fig. 1:**
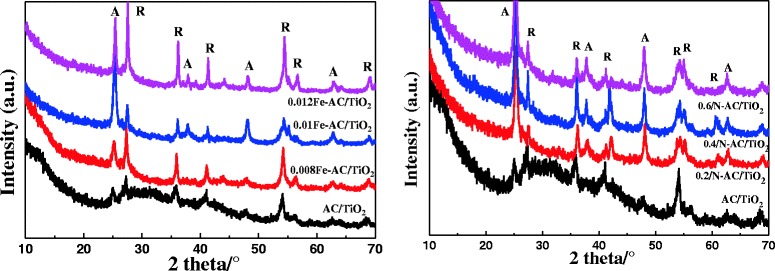
XRD patterns of undoped, Fe-doped, and N-doped TiO_2_. *A* refers to the anatase crystal, *R* refers to the rutile crystal

To determine the optimal concentration of Fe and N for codoping, AC/TiO_2_ was monodoped with Fe or N at three different concentrations. The XRD was used to investigate the composition of the crystalline phase and the average size of the catalysts. Anatase and rutile phase that are commonly existed in all samples can be seen in Fig. 1. The peaks observed at 25.3°, 38°, and 48° represent the anatase crystalline phase [14], whereas the peaks at 27.42°, 36.2°, 41.3°, 44.2°, 54.45°, and 56.82° represent the rutile crystalline phase. The diffraction peaks of anatase phase (101) widened, and their intensity strengthened with Fe and N doping, indicating lower crystallinity. As previously reported [16], both Fe and N influence crystallite growth, ratio of anatase phase to rutile phase, and mean crystallite sizes. The crystallite sizes of the samples were calculated using the Scherrer equation from the full widths at half maximum of the anatase (101) and rutile (110) peaks (Table 1). The results showed that the particle sizes of Fe-AC/TiO_2_ are larger than those of AC/TiO_2_, likely because Fe^3+^ occupies the position of Ti^4+^ in TiO_2_ and distorts the crystal structure of the host compound owing to the difference in atomic size between Fe^3+^ (0.079 nm) and Ti^4+^ (0.075 nm). Although the crystallite size of N-doped samples was similar to that of undoped samples, their anatase content increased because of N doping (Table 1). The ionic radius of N^3−^ (0.171 nm) is much larger than that of O^2−^ (0.144 nm). This difference in size induces the N atoms to lock the Ti–O species at the interface with the TiO_2_ domains, thereby preventing anatase to rutile phase transformation when O^2−^ is substituted by N^3−^ in the unit cell. In this study, however, no other peak besides that of TiO_2_ was detected, which may be because the low concentration of Fe or N in the composition and sol–gel process allows uniform distribution of the dopants to form a solid solution.

**Table 1:** Powders with different dopant concentrations

Samples	Anatase size (nm)	Rutile size (nm)	Ratio of A and R %
AC/TiO_2_	17.9	22.9	47/53
0.008 Fe-AC/TiO_2_	19.6	24.1	49/51
0.01 Fe-AC/TiO_2_	21.6	25.8	59/41
0.012 Fe-AC/TiO_2_	32.4	39.3	33/67
0.2 N-AC/TiO_2_	15.4	11.5	72/28
0.4 N-AC/TiO_2_	17.2	18.6	62/38
0.6 N-AC/TiO_2_	18.8	16.3	69/31

**Fig. 2:**
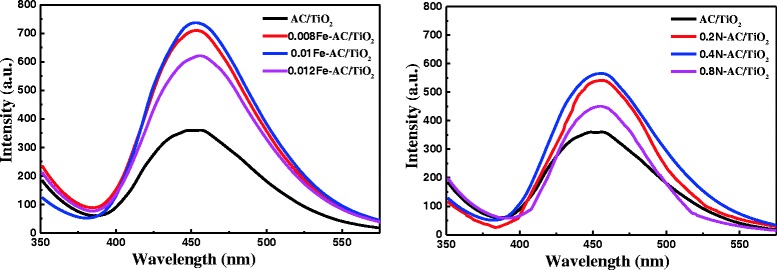
PL spectra of undoped, Fe-doped, and N-doped AC/TiO_2_ powders with different dopant concentrations

According to earlier studies [26], the photocatalytic activity of the photocatalysts could be partially attributed to the amount of ⋅OH induced in the reaction system. The ⋅OH generated on the surface of different photocatalysts was determined using PL emission spectra. Figure 2 shows the of PL spectra from 5 × 10^−4^ mol/L terephthalic acid solution in 2 × 10^−3^ mol/L NaOH under Xe lamp irradiation in the presence of AC/TiO_2_, Fe-AC/TiO_2_, and N-AC/TiO_2_ photocatalysts. It is clear that the PL spectra of the photocatalysts have a strong emission peak at around 450 nm. The rate of ⋅OH radical generation on the AC/TiO_2_ surface was lower than that on the other photocatalysts, which indicates that Fe doping changes the crystal structure and optical properties of the photocatalysts. The anatase/rutile ratio is an important factor determining the photocatalytic activity of a photocatalyst through ⋅OH formation [28, 29]. The rate of ⋅OH formation by Fe-AC/TiO_2_ is enhanced by the increase in anatase/rutile ratio with increasing amount of Fe. The highest rate of ⋅OH formation was observed when the amount of Fe was 0.01 g (Fig. 2). Further increases in the amount of Fe to 0.012 g retained the composite structure of the sample with an anatase/rutile ratio of 33:67 but decreased the formation rate of ⋅OH. With N-AC/TiO_2_, the anatase content significantly increased because of nitrogen introduction, and 0.4 N-AC/TiO_2_ showed the highest ⋅OH formation rate. It can be seen that the photocatalyst showed higher ⋅OH formation rate when the anatase/rutile ratio was about 60:40.

**Fig. 3:**
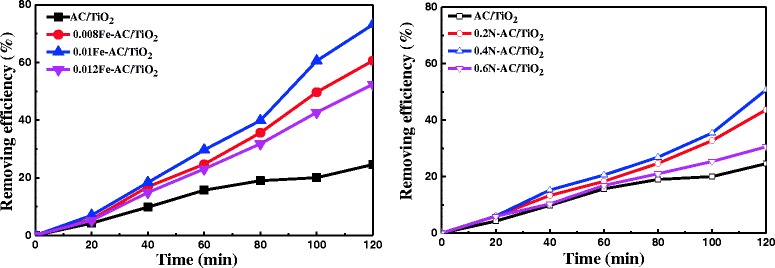
Effect of Fe/N doping content in AC/TiO_2_ on HCHO degradation. ([HCHO] = 30 mg/L, volume of HCHO = 50 mL, photocatalyst amount = 50 mg)

HCHO degradation was examined in the presence of undoped and doped AC/TiO_2_ powders under Xe-lamp irradiation to determine the resulting photocatalytic responses. Regardless of the doping concentration, N- and Fe-doped AC/TiO_2_ showed higher photocatalytic activities than undoped AC/TiO_2_ (Fig. 3). Moreover, Fe-doped TiO_2_ exhibited considerably better activity than N-doped TiO_2_. This finding is consistent with the results of Li et al. [24] reported that the rate of degradation of methyl orange by Fe/TiO_2_ is higher than that of N/TiO_2_ under natural light exposure for 60 min. At low Fe content (≤0.01 g), the photocatalytic activity of Fe-AC/TiO_2_ gradually increased with increasing Fe content; the best performance was observed when the Fe content was 0.01 g. However, when the Fe content was increased to 0.012 g, the photocatalytic activities of the products decreased. The photodegradation performance of N-AC/TiO_2_ was similar to that of Fe-AC/TiO_2_, and 0.4 N-AC/TiO_2_ showed the highest photocatalytic activity. These results are in accordance with the PL intensities observed (Fig. 2). On the basis of these results, dopant concentrations of 0.01 g for Fe and 0.4 g for N were selected for codoping for further studies. Henceforth, the codoped sample is designated as 0.4 N-0.01 Fe-AC/TiO_2_.

**Fig. 4:**
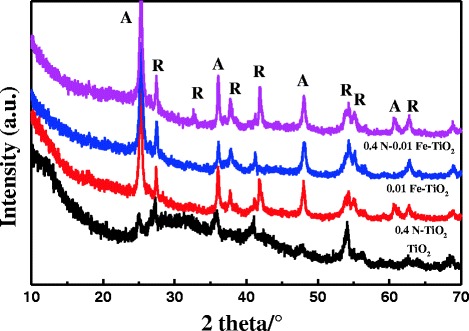
XRD patterns of undoped, 0.01 Fe-doped, 0.4 N-doped, and 0.01 Fe–0.4 N-codoped AC/TiO_2_ powders

**Fig. 5:**
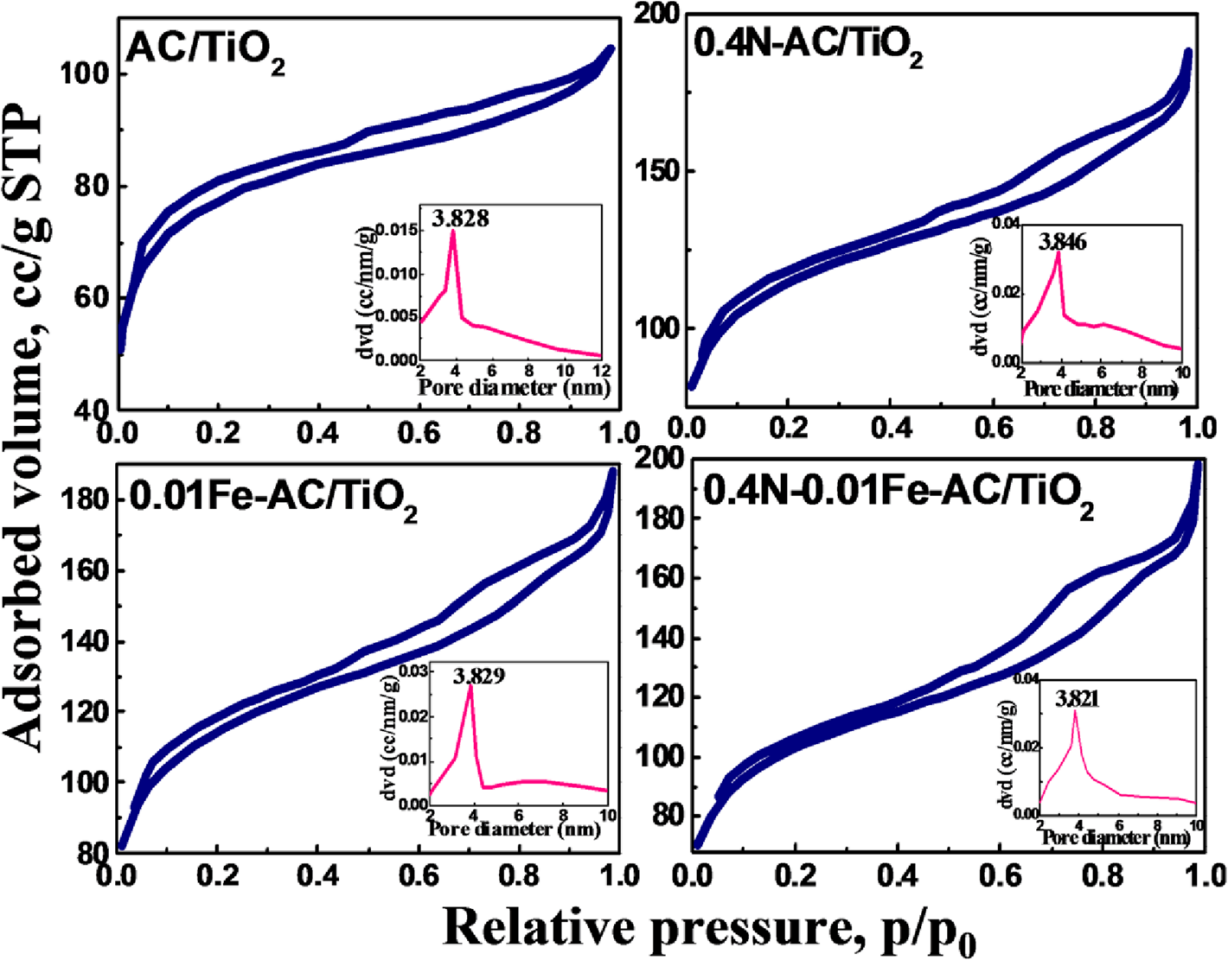
Nitrogen adsorption-desorption isotherms and pore size distribution curves (inset) of undoped, 0.4 N-doped, 0.01 Fe-doped, and 0.4 N-0.01 Fe-codoped AC/TiO_2_ powders

Figure 4 shows the X-ray diffraction (XRD) patterns of the codoped AC/TiO_2_ catalysts, revealing that the bulk of the 0.4 N-0.01 Fe-AC/TiO_2_ powder contains both anatase and rutile phases. The width of the (101) plane diffraction peak of anatase became narrower than that of monodoped AC/TiO_2_. In codoped TiO_2_, Fe^3+^ replaces Ti^4+^, and the size difference produces strain energy by lattice distortion. N^3−^ also replaces O^2−^ ions, thereby creating oxygen deficiencies in the TiO_2_ lattice. These changes allow the rearrangement of Ti^4+^ and O^2−^ ions in the lattice to favor anatase to rutile phase transformation [14]. Surface area measurements obtained by the single-point Brunauer–Emmett–Teller method using N_2_ absorption are shown in Fig. 5. According to the IUPAC classification, the N_2_ adsorption isotherms of the samples are of type IV, with a type-H_4_ hysteresis loop. Degradation of organic pollutants generally occurs on the surface of AC in the AC/TiO_2_ system; thus, the specific surface area of the catalyst plays an important role in this process. In this study, the specific surface area and pore size of AC (Fig. 5, inset) did not significantly change after the ions were codoped in AC/TiO_2_ (Table 2).

**Table 2:** Physicochemical properties of undoped, mono-doped and co-doped AC/TiO_2_ powders

Samples	Anatase size (nm)	Rutile size (nm)	SBET (m^2^/g)	Band gap (eV)	Ratio of A and R %
AC/TiO_2_	17.9	22.9	532	2.86	47/53
0.4 N-AC/TiO_2_	17.2	18.6	487	2.81	62/38
0.01 Fe-AC/TiO_2_	21.6	25.8	562	2.79	59/41
0.4 N-0.01 Fe-AC/TiO_2_	19.3	23.4	550	2.58	57/43

**Fig. 6:**
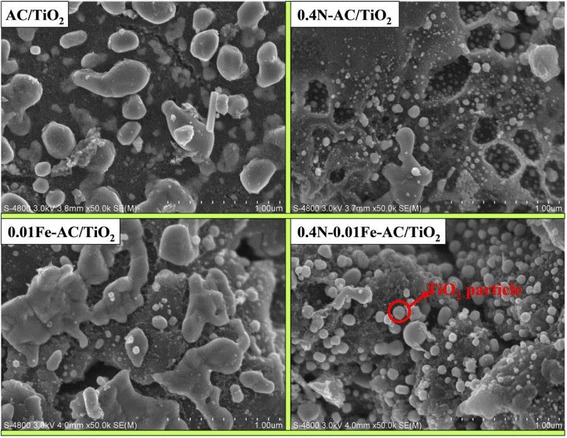
SEM images of undoped, 0.4 N-doped, 0.01 Fe-doped, and 0.4 N-0.01 Fe-codoped AC/TiO_2_ powders

**Fig. 7:**
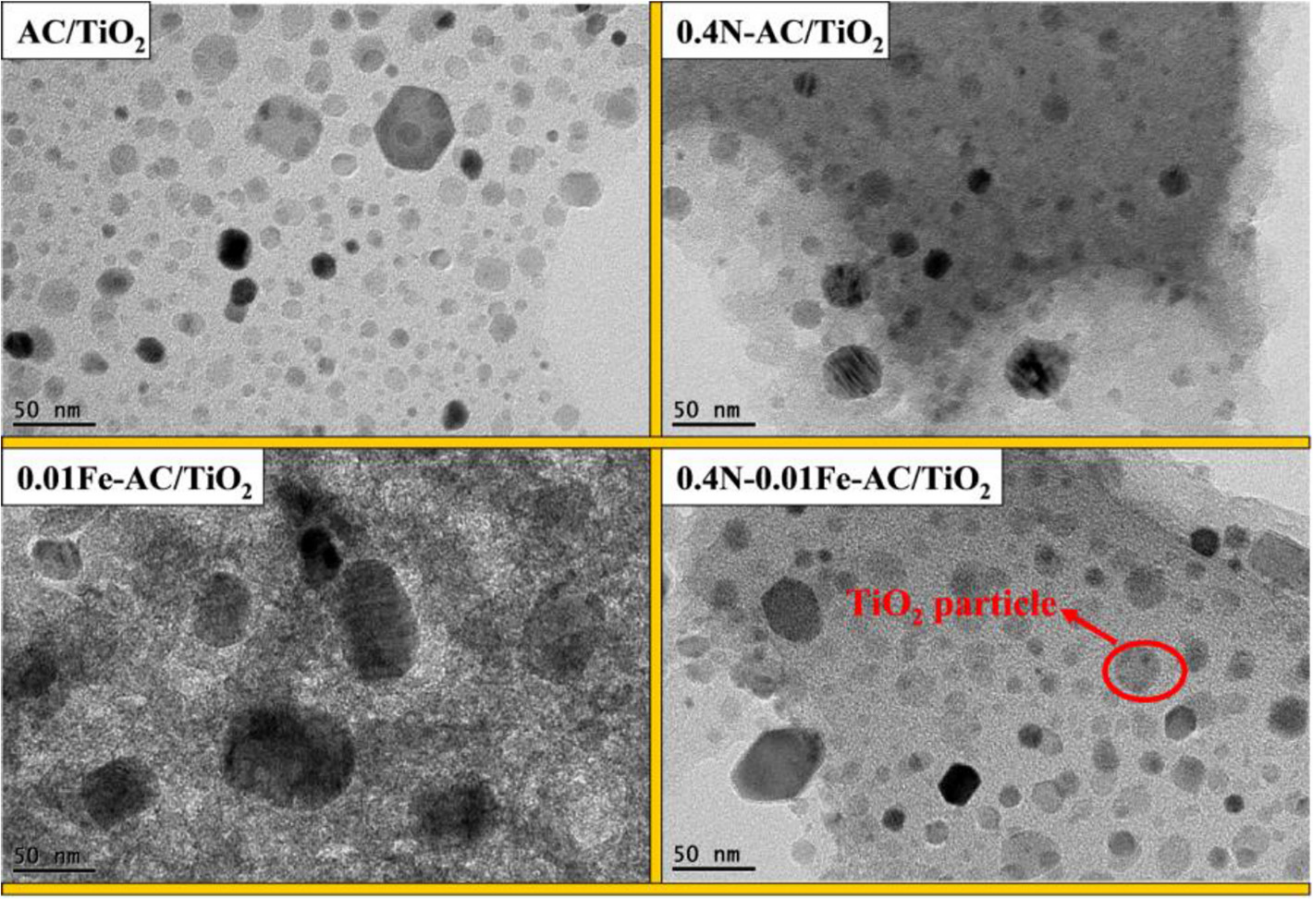
TEM images of undoped, 0.4 N-doped, 0.01 Fe-doped, and 0.4 N-0.01 Fe-codoped AC/TiO_2_ powders

Figure 6 presents SEM micrograph of the updoped AC/TiO_2_, 0.4 N-AC/TiO_2_, 0.01 Fe-AC/TiO_2_, and 0.4 N-0.01 Fe-AC/TiO_2_ photocatalyst. It has been observed that nano-TiO_2_ particles were obtained rapidly via microwave heating method and are uniformly dispersed on the AC surface in all samples. Especially, the TiO_2_ particles in the codoped samples exhibit uniform, dense, and compact morphologies. The morphology and microstructure of the samples were further analyzed using TEM (Fig. 7). Undoped AC/TiO_2_ exhibited a spherical morphology, with average particle sizes of ~23 nm. 0.4 N-AC/TiO_2_ also presented a spherical morphology but had a smaller particle size (~18 nm) than undoped AC/TiO_2_. Conversely, large crystallites of 26 nm were observed for 0.01 Fe-AC/TiO_2_. The TiO_2_ particles of 0.4 N-0.01 Fe-AC/TiO_2_ were spherical with particle sizes of ~20 nm. The particle sizes obtained through TEM analysis were in good agreement with the result calculated by XRD in Table 1.

**Fig. 8:**
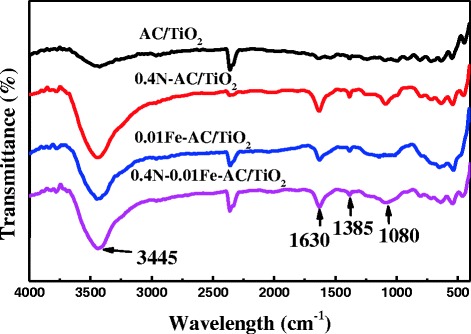
FTIR spectra of undoped, 0.4 N-doped, 0.01 Fe-doped, and 0.4 N-0.01 Fe-codoped AC/TiO_2_ powders

Figure 8 shows FTIR spectra of AC/TiO_2_, 0.4 N-AC/TiO_2_, 0.01 Fe-AC/TiO_2_, and 0.4 N-0.01 Fe-AC/TiO_2_. The strong peak observed at 3445 cm^−1^ can be assigned to the bending vibration of -OH or H_2_O on the photocatalyst [24], and the vibrations of the hydroxyl moiety were enhanced upon AC/TiO_2_ doping. Kim et al. [17] reported that photoinduced holes can attack surface hydroxyl groups and yield surface ⋅OH with high oxidation capability. Thus, the doped AC/TiO_2_ may have better photocatalytic activity than undoped AC/TiO_2_. The absorption peak at 1630 cm^−1^ corresponded to the Ti–O structure [30], and the absorption peak at 1385 cm^−1^ was assigned to the C–H species. Compared with AC/TiO_2_ and Fe-AC/TiO_2_, N-AC/TiO_2_ and N-Fe-AC/TiO_2_ displayed additional peaks at approximately 1080 cm^−1^, which can be assigned to the vibration of the N–Ti bond formed when N atoms are embedded in the TiO_2_ network [20]. Fe-O-Ti at 570 cm^−1^ was not observed, which may be ascribed to its low doping content and high dispersion. The peak from 750 to 400 cm^−1^ bands are related to the bend vibration of Ti-O-Ti bonds [2].

**Fig. 9:**
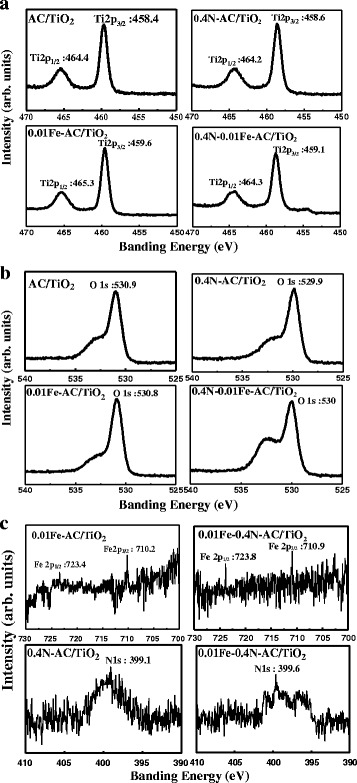
XPS spectra of undoped, 0.4 N-doped, 0.01 Fe-doped, and 0.4 N-0.01 Fe-codoped AC/TiO_2_ powders: (**a**) Ti2p, (**b**) O1s, (**c**) Fe2p and N1s

The XPS analysis (Fig. 9) was performed in order to characterize elemental chemical states in undoped AC/TiO_2_, 0.4 N-AC/TiO_2_, 0.01 Fe-AC/TiO_2_, and 0.4 N-0.01 Fe-AC/TiO_2_. The binding energies of all of the samples at approximately 459 and 464 eV may be assigned to 2p_3/2_ and 2p_1/2_ of Ti, respectively, which indicates that Ti ions are in an octahedral environment [23] and that their valence state (+4) is not influenced by a small amount of doping. However, the binding energy of Ti 2p_3/2_ of doped AC/TiO_2_ is larger than that of undoped AC/TiO_2_ because of the interaction of ions with TiO_2_, which suggests that the surface acidity of TiO_2_ is enhanced and that polar organic pollutants easily adsorb on the catalyst surface [30]. The peaks at approximately 530 eV in the O1s region correspond to the Ti–O bond in TiO_2_ and the surface hydroxyl groups of TiO_2_ [17]. Upon examination of the Fe2p core level, two peaks at 710 and 723 eV, corresponding to the binding energies of Fe 2p_3/2_ and Fe 2p_1/2_, respectively, of Fe_2_O_3_ for both 0.01 Fe-AC/TiO_2_ and 0.4 N-0.01 Fe-AC/TiO_2_ samples, may be observed [31]. The Fe2p peaks reveal weak intensity resulting from the low doping level. Because the ionic radii of Fe^3+^ and Ti^4+^ are similar, Fe^3+^ could be incorporated into the lattice of TiO_2_ to form Ti–O–Fe bonds in the 0.01 Fe-AC/TiO_2_ and 0.4 N-0.0.1 Fe–AC/TiO_2_ samples [17]. The peak at approximately 399 eV was assigned to N1s in the 0.4 N-AC/TiO_2_ and 0.4 N-0.01 Fe-AC/TiO_2_ lattices. The N1s peak at approximately 400 eV can be attributed to the presence of oxidized N, such as Ti–O–N or Ti–N–O binding, so the peaks at 399.1 and 399.6 eV can be attributed to the anionic N^−^ in the interstitial N form. Asahi et al. [32] pointed out that N2p states can mix with O2p states, and thus, N atoms can substitute O atoms in the TiO_2_ crystal lattice. The XPS results demonstrate that Fe and N were successfully codeposited into the TiO_2_ lattice of 0.4 N-0.01 Fe-AC/TiO_2_.

**Fig. 10:**
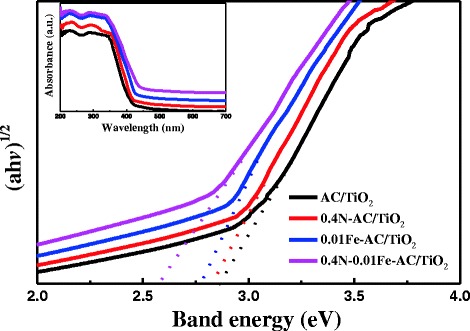
Diffuse reflectance UV–vis spectra of undoped, 0.4 N-doped, 0.01 Fe-doped, and 0.4 N-0.01 Fe-codoped AC/TiO_2_ powders

The UV–vis absorption spectra of prepared AC/TiO_2_, 0.4 N-AC/TiO_2_, 0.01 Fe-AC/TiO_2_, and 0.4 N-0.01 Fe-AC/TiO_2_ photocatalysts are shown in Fig. 10 inset. In general, the fundamental absorption edge of elemental doped TiO_2_ can redshift toward the visible light region. This phenomenon is more apparent in codoped AC/TiO_2_ than in N- and Fe-doped AC/TiO_2_ in previous studies. TiO_2_ is known as an indirect semiconductor, for which the Kubelka–Munk function between the absorption coefficient (*a*) and the incident photon energy (h*ν*) can be written as *a* = *B*i(h*ν*-*E*_*g*_)^2^/h*ν*, where *B*i is the absorption constant for indirect transitions, h*v* is the photon energy, and *E*_*g*_ is the band gap energy. Plots of (*a*h*ν*)^1/2^ versus h*ν* from the spectral data are presented in Fig. 10. After monodoping, the band gap value decreased from 2.86 eV (AC/TiO_2_) to 2.81 and 2.79 eV for 0.4 N- and 0.01 Fe-AC/TiO_2_ powders, respectively. However, for the codoped powder (0.4 N-0.01 Fe-AC/TiO_2_), the absorption edge decreased continuously in the lower energy range (2.58 eV). This behavior, previously reported in different doped TiO_2_ nanostructures, is usually interpreted as the result of the introduction of new intra-gap energy levels [33]. For N-AC/TiO_2_, the N that enters into the TiO_2_ lattice provides a new occupied orbital between the valence band (VB) of the O2p orbital and the conduction band (CB) of the Ti3d orbital, forming new energy levels between the VB and the CB within the band gap of TiO_2_ [22]. Electrons from the original VB can migrate into the mid-band gap energy level, leaving a hole in the VB. Xing et al. [34] showed that N doping effectively reduces the band gap of TiO_2_ by generating an isolated N2p narrow band above the O2p valence, which is formed by incorporation of N atoms into the TiO_2_ lattice. When Fe^3+^ was doped into AC/TiO_2_, a new energy level was formed below the CB of TiO_2_. The Fe^3+^ ion can become the trapping site of photoinduced electrons because of the reducibility of these electrons, thereby reducing Fe^3+^ to Fe^2+^. Fe^2+^ ions then become the trapping site of holes, as holes feature oxidizability [10]. These findings suggest that N and Fe codoping of AC/TiO_2_ exerts a synergistic effect on reducing the band gap. Band gaps in semiconductor materials are closely related to the wavelengths they absorb and decrease with increasing absorption wavelength. Therefore, compared with N- and Fe-doped AC/TiO_2_, codoped AC/TiO_2_ may be expected to be a more active photocatalyst.

**Fig. 11:**
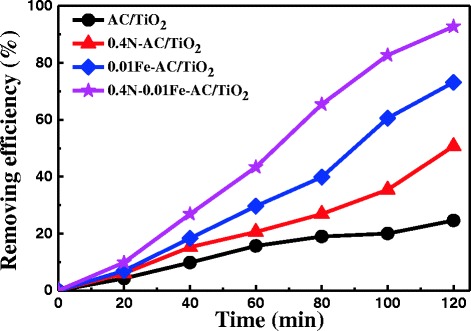
Comparison of photocatalytic degradation of HCHO under light irradiation in presence of undoped, 0.4 N-doped, 0.01 Fe-doped, and 0.4 N-0.01 Fe-codoped AC/TiO_2_ ([HCHO] = 30 mg/L, volume of HCHO = 50 mL, photocatalyst amount = 50 mg)

**Fig. 12:**
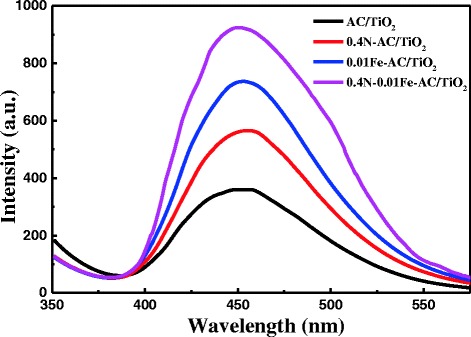
Photoluminescence emission spectra of undoped, 0.4 N-doped, 0.01 Fe-doped, and 0.4 N-0.01 Fe-codoped AC/TiO_2_ powders

The photocatalytic activity of 0.4 N-0.01 Fe-AC/TiO_2_ was compared with the monodoped (0.4 N-AC/TiO_2_ and 0.01 Fe-doped AC/TiO_2_) and undoped-AC/TiO_2_ for photodegradation of HCHO (Fig. 11). The codoped AC/TiO_2_ samples showed higher photocatalytic efficiency than other photocatalysts surveyed during the degradation process. Approximately 93 % of the available HCHO was degraded in 120 min. The most important factor influencing the observed enhanced catalytic activity is the ability of the doped AC/TiO_2_ to absorb large amounts of visible light and produce ⋅OH. The PL spectra of 0.4 N-0.01 Fe-AC/TiO_2_ were compared with those of the monodoped and undoped AC/TiO_2_ photocatalysts (Fig. 12). The generation rate of ⋅OH radicals on the 0.4 N-0.01 Fe-AC/TiO_2_ surface was higher than those of the other photocatalysts, consistent with the photocatalytic efficiency illustrated in Fig. 11.

Several factors may result in the high generation rate of ⋅OH radicals and photocatalytic activity. First, codoped AC/TiO_2_ contains anatase (57 %) and rutile (43 %) phases, which reduce the recombination of photogenerated electrons and holes and enhance the formation rate of ⋅OH [28]. Electrons from the VB can be excited and moved to the CB of TiO_2_ by photon absorption. In undoped AC/TiO_2_, the band gap energy between the VB and CB of TiO_2_ is 3.86 eV. Fe ion occupancy in the Ti sites of the TiO_2_ lattice of Fe-AC/TiO_2_ can be seen in the XPS results shown in Fig. 9. When the catalysts were subjected to solar irradiation, the 3d electrons of Fe^3+^ were excited into the CB of TiO_2_, which introduces a new energy level [31]. Electrons can be excited in two stages; the first stage involves electron excitation from lower new states to the CB photon adsorption and the second stage involves excitation from the VB to the lower Fe^3+^ states. Thus, an interaction among the d electrons of Fe and the TiO_2_ CB or VB occurs, eventually narrowing the energy gap of TiO_2_ through the formation of new intermediate energy levels. In N-AC/TiO_2_, the Ti–N linkage (Fig. 8) is believed to lead to the formation of the N1s peak (Fig. 9), which is due to substitutional N doping in the TiO_2_ lattice. Doping of N into TiO_2_ forms a new state on N1s just above the O2p VB, leading to the strong absorption of visible light and enhancing the separation efficiency of photoinduced electrons and holes. Fe^3+^ and N doping can suppress the recombination rate of electron–hole pairs and improve the photocatalytic activity of the resultant catalysts. Therefore, the cooperation of Fe^3+^ and N induces the formation of new energy levels close to the CB and VB, respectively, leading to a much narrower band gap and greatly improved photocatalytic activity.

**Fig. 13:**
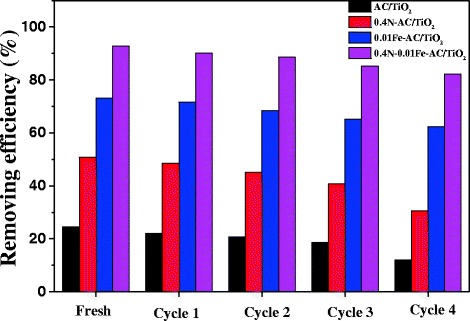
Repetitive use of undoped, 0.4 N-doped, 0.01 Fe-doped, and 0.4 N-0.01 Fe-codoped AC/TiO_2_ photocatalyst ([HCHO] = 30 mg/L, volume of HCHO = 50 mL, photocatalyst amount = 50 mg)

To confirm the stability and durability, the photocatalytic performances of 0.4 N-AC/TiO_2_, 0.01 Fe-AC/TiO_2_, and 0.4 N-0.01 Fe-AC/TiO_2_ under visible light for the degradation of HCHO were investigated in four cycles (Fig. 13). The photocatalysts exhibited no apparent decrease in photocatalytic degradation of HCHO after four cycles of reuse. Among the samples surveyed, 0.4 N-0.01 Fe-AC/TiO_2_ showed the best photochemical stability, degrading 83 % of the available HCHO within 120 min even after four recycles. These results indicate that the photocatalysts present excellent stability and durability for practical applications.

## Conclusion

N-Fe-AC/TiO_2_ photocatalyst was successfully synthesized through an efficient and rapid microwave-assisted sol–gel method. The sphere-like TiO_2_ showed anatase and rutile phases with a particle size of 20 nm in codoped AC/TiO_2_, which features a specific surface area of 550 m^2^/g. N and Fe ions occupied the TiO_2_ lattice, replacing some Ti^4+^ and O^2−^, respectively, and extending the absorption range of the catalyst to the visible light region. The N-Fe-AC/TiO_2_ photocatalyst exhibited better photocatalytic activity than undoped and Fe/N-monodoped AC/TiO_2_, which degraded 93 % of HCHO within 120 min under Xe-lamp irradiation. N-Fe codoping may have induced the formation of new states between the VB and CB. Moreover, N-Fe codoping can promote the separation of photogenerated electrons and holes to accelerate the transmission of photocurrent carriers. The produced photocatalyst can be easily recycled, which reveals its enhanced stability. These results suggest that the prepared (N, Fe)-codoped AC/TiO_2_ exhibits the characteristics of a highly effective photocatalyst under visible light irradiation.
